# Changes in the Aqueous Solvent do not Impact the Internal Ring‐Flip Dynamic of Fully Buried F52 in Protein GB1

**DOI:** 10.1002/cbic.202500183

**Published:** 2025-06-04

**Authors:** Matthias Dreydoppel, Mikhail Achkinazi, Charlotte Krünholz, Paula L. Jordan, Ulrich Weininger

**Affiliations:** ^1^ Institute of Physics, Biophysics Martin‐Luther‐University Halle‐Wittenberg D‐06120 Halle (Saale) Germany; ^2^ Department of Radiology, Medical Physics University Medical Center Freiburg Faculty of Medicine University of Freiburg D‐79106 Freiburg Germany

**Keywords:** NMR spectroscopies, protein breathings, protein dynamics, protein hydrations, water dynamics

## Abstract

Aromatic ring flips are a hallmark of protein dynamics. They are mediated by either transient “breathing” motions in which the protein expands into the solvent or by transient internal rearrangement of void spaces. Therefore, they are excellent reporters of such transient protein fluctuations. To decipher the extent to which ring‐flip dynamics are governed by the protein itself or by the aqueous solvent around it, the ring flip of the fully buried aromatic side chain of F52 in protein B1 domain of immunoglobulin G binding protein G(GB1) with experimentally feasible altered buffer conditions by nuclear magnetic resonance relaxation dispersion experiments is studied. Herein, it is found that ring‐flip rate constants remain the same in all studied cases. Therefore, the ring‐flip dynamic in the interior of GB1 is independent from the solvent and only depends on the protein itself. In addition, this study shows that ring flips are comparable within different buffer conditions.

## Graphical abstract

The ring‐flip dynamic in the interior of a protein is independent from changes in the solvent and only depends on the protein itself.

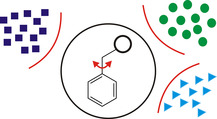

## 1 Introduction

Proteins are structural and dynamic molecules. It is well established that both structure^[^ ^1^ ^]^ and dynamics^[^ ^2^, ^3^, ^4^ ^]^ are essential for their biological function. What is less clear and an ongoing debate is, to what exact extent the aqueous solvent, and its inherent dynamics, impacts the dynamics of a protein.^[^ ^5^, ^6^, ^7^, ^8^ ^]^ In the extreme case, protein dynamics would be just slaved to existing water dynamics.^[^ ^9^ ^]^ Fast timescale water dynamics are coupled to fast timescale dynamics of the protein surface and can propagate into slower dynamics and the interior of the protein, like in protein folding or a ligand entering or leaving the inside of a protein.

Aromatic residues contribute significantly (roughly 25% of the volume in average) to the hydrophobic core of a protein. They stabilize a protein by the so‐called hydrophobic effect, where hydrophobic side chains are excluded from the aqueous solvent,^[^ ^10^, ^11^ ^]^ and by their quadrupolar electrostatic character, which allows them to form specific aromatic–aromatic interactions^[^ ^12^, ^13^ ^]^ and interactions with sulfur^[^ ^14^ ^]^ or cations.^[^ ^15^ ^]^ In addition, symmetric Phe and Tyr residues undergo frequent 180° rotations of the *χ*_2_ dihedral angle (around the imaginary C_β_‐C_γ_‐C_ζ_ axis), so‐called ring flips, which makes them unique reporters of fundamental and transient dynamic processes in proteins.^[^ ^16^, ^17^, ^18^ ^]^ In order for a ring flip to occur, interactions near the ring have to be broken and atoms have to move in a concerted way to create the required void volume. Accordingly, the transition state of a ring flip displays an increase in enthalpy, reflecting broken contacts, and an increase in entropy, reflecting a loss of structural order.^[^ ^17^, ^19^, ^20^, ^21^, ^22^, ^23^, ^24^ ^]^ The transition state is liquid‐like in contrast to the tightly packed solid‐like ground state.^[^ ^25^ ^]^ Furthermore, the transition state often displays an increased volume of the protein (“breathing motions”),^[^ ^19^, ^20^, ^25^, ^26^, ^27^ ^]^ although it is possible but less efficient to create the necessary void volume without expansion of the protein, by internal rearrangement of voids.^[^ ^25^ ^]^

Ring flips can be studied by MD simulations and experimentally by nuclear magnetic resonance (NMR) spectroscopy. MD is perfectly suited for studying fast ring flips, while slowed down ring flips, which are usually found in structured regions and the interior of proteins, can only be studied by very long or accelerated MD simulations, in certain cases. In contrast, NMR is perfectly suited for studying such slowed down ring flips, using site‐selective isotope labeling^[^ ^28^, ^29^, ^30^, ^31^, ^32^, ^33^, ^34^, ^35^, ^36^ ^]^ and state‐of‐the‐art ^1^H‐ and ^13^C‐based relaxation dispersion methods for aromatic side chains.^[^ ^37^, ^38^, ^39^, ^40^, ^41^ ^]^

**Figure 1:**
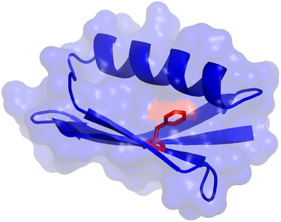
3D structure of GB1. Secondary structures and surface are shown in blue. F52 is shown as sticks in red.

Since ring‐flip dynamics are somewhat intermediate in timescale and the number of atoms involved, and are associated with an expansion of the protein in the solvent, the question arises how dependent the ring‐flip dynamic is on the solvent. Here we investigate the slow ring flips of F52 in B1 domain of immunoglobulin G binding protein G (GB1) in dependence of variations within the aqueous solvent. F52 is the central residue of an aromatic cluster (**Figure** **1**), with close to no solvent accessibility (4 Å^2^). We change the charge composition of GB1 by altering pH and vary the osmotic strength and the surface tension by use of two different salts. Finally, we increase crowding and viscosity by variation of two different macromolecular crowders. None of these variations of the aqueous solvent changes the activation energy (or the ring‐flip rate constants) of the ring flip of F52, within errors. Thus, there is no general impact of variations of the solvent on the ring‐flip dynamics in the interior GB1. Furthermore, activation enthalpy and entropy are also unchanged, within errors, pointing to no visible enthalpy–entropy compensation. Ring‐flip dynamics in the interior of GB1 depend only on the structural and energetic aspects of the protein itself and not on variations of the solvent. In addition, determined rate constants of ring flips can be directly compared despite possible small variations in the solvent conditions.

## 2 Results and Discussion

F52, the central residue of the aromatic cluster in GB1 (Figure 1), undergoes fast ring‐flip dynamics. This ring‐flip dynamic is well studied^[^ ^19^, ^25^, ^40^, ^42^ ^]^ and significantly affected by temperature and pressure. So far, all of these studies have been conducted in 20 mM 4‐(2‐hydroxyethyl)‐1‐piperazineethanesulfonic acid (HEPES) buffer, pH 7.0. The other residues in GB1, that undergo slow ring flips, Y3, F30, and Y45, are solvent accessible and display lower quality data, which has to be obtained under different conditions. Therefore, they are not included in this study.

### 2.1 The Ring‐Flip Dynamic of F52 does not Depend on Solvent‐Induced Changes in the Protein Electrostatics

**Figure 2:**
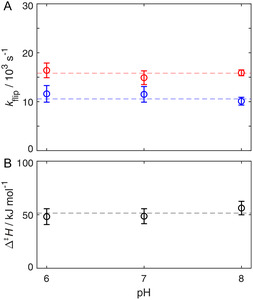
A) pH dependence of F52 ring‐flip rates. *k*_flip_ is plotted as a function of pH at temperatures of 15 °C (blue) and 20 °C (red). B) Activation enthalpies for different pH values. Δ^‡^ *H* values were derived from flip rates at temperatures ranging from 10 to 20 °C using Equation (1). Dashed lines represent linear fits with zero slope of the respective data points to guide the eye.

**Figure 3:**
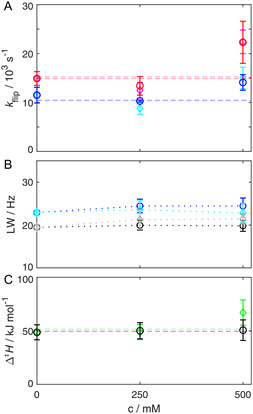
A) Salt concentration dependence of F52 ring‐flip rates. *k*_flip_ is plotted as a function of the concentration of NaI at temperatures of 15 °C (blue) and 20 °C (red) and of Na_2_SO_4_ at 15 °C (cyan) and 20 °C (magenta), respectively. B) ^13^C linewidths for different concentrations of NaI and Na_2_SO_4_ (F52ε blue and cyan, F30ε black and grey, respectively), at 15 °C. C) Activation enthalpies for different concentrations of NaI (black) and Na_2_SO_4_ (green). Δ^‡^ *H* values were derived from flip rates at temperatures ranging from 10 to 20 °C using Equation (1). To guide the eye, dashed lines in A and C represent linear fits with zero slope and dotted lines in B are simple links of the respective data points.

To investigate a possible impact of the buffer on the ring‐flip dynamic of the interior of GB1, we studied the ring flip of F52 between pH 6 and pH 8. There are no histidines in GB1, but D37 displays a p*K*_a_ value of 6.5, meaning its protonation and charge changes significantly in this pH range. It is located remotely from the aromatic cluster at the C terminus of the helix. In addition, other Asp and Glu residues become protonated at pH 6.^[^ ^43^ ^]^ All in all, the conducted changes in pH affect the electrostatics of GB1, without interfering directly with F52 and its surrounding. The ^13^C *R*_1ρ_ relaxation dispersion derived ring‐flip rate constants of F52 do not change with pH, exemplified for 15 and 20 °C (**Figure** **2**A and S1, Supporting Information). Furthermore, there is no change in the activation enthalpy (Figure 2B), indicating that there is no enthalpy–entropy compensation. So, the findings should hold at other temperatures as well. To further elaborate the possible impact of electrostatics, we measured a salt dependence of the ring‐flip dynamic going up to 500 mM NaI and Na_2_SO_4_. Both salts display a similar osmotic strength.^[^ ^44^ ^]^ Again, ^13^C *R*_1ρ_ relaxation–dispersion‐derived ring‐flip rate constants and activation enthalpy do not change within errors (**Figure** **3** and S2, Supporting Information). However, high salt leads to significant performance losses in the NMR experiments, by increasing ^1^H pulse lengths and decreasing the signal to noise ratio (S/N). This becomes visible in the increased error bars at 500 mM salt (Figure 3). To further verify the finding that salt does not alter the ring‐flip dynamic we analyzed the ^13^C linewidths of F52ε, that are impacted by ring‐flip dynamics, and F30ε, which is unaffected by ring flips, since the chemical shift difference for both sides of the ring is zero (Figure 3B). In contrast to relaxation dispersion derived ring‐flip rate constants, this analysis is only qualitative in this instance (broader linewidth—slower ring flips, narrower linewidth faster ring flips), but it reveals the same overall result: there is no effect on the ring‐flip dynamic in the interior of a protein by salt concentrations up to 500 mM. Quantitative ring‐flip studies at higher salt concentrations would be extremely challenging. Taken together, there is no clear correlation between moderate changes in pH and salt concentration of the aqueous solvent, and consequently changes in protein electrostatics, and the ring‐flip dynamic in the interior of a protein. This implies that flip dynamics of rings which are not solvent accessible are comparable under different buffer conditions.

### 2.2 An Increase of Surface Tension does not Have an Impact on Ring‐Flip Dynamics

NaI and Na_2_SO_4_ not only increase the osmotic strength of the aqueous solvent but also increase its surface tension by affecting the hydration properties of the protein solution. Na_2_SO_4_ increases the surface tension by more than a factor of two for the same concentration in comparison to NaI.^[^ ^44^ ^]^ Na_2_SO_4_ generally favors the reduction of the exposure of protein surface to the solvent, whereas NaI can contribute to the exposure of protein surface. Since the transition state of a ring flip is usually expanded compared to its ground state, one does expect a lower ring‐flip rate constant with increased surface tension in close analogy to the effect of increased hydrostatic pressure.^[^ ^19^, ^20^, ^25^, ^26^, ^27^ ^]^ This is clearly not the case (Figure 3). Moreover, the slight increase in the rate constant of the ring flips at 500 mM salt, which goes together with an increased experimental error, is not connected to surface tensions (even in a contradictory direction), since the surface tension of a 250 mM Na_2_SO_4_ solution is higher than the surface tension of a 500 mM NaI solution.^[^ ^45^ ^]^ In summary, the increase of surface tension of the solvent by salt is too small to reflect in visibly slowed down ring flips, as it is in contrast seen at high hydrostatic pressure.

### 2.3 A Crowded Environment in the Solvent does not Slow Down Ring‐Flip Dynamics

**Figure 4:**
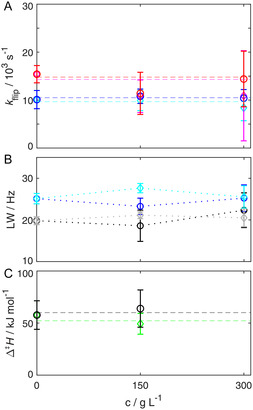
A) Crowder concentration dependence of F52 ring‐flip rates. *k*_flip_ is plotted as a function of the concentration of PEG at temperatures of 15 °C (blue) and 20 °C (red), and of dextran at 15 °C (cyan) and 20 °C (magenta), respectively. B) ^13^C linewidths for different concentrations of PEG and dextran (F52ε blue and cyan, F30ε black and grey, respectively), at 15 °C. C) Derived activation enthalpies for different concentrations of PEG (black) and dextran (green). Δ^‡^ *H* values were derived from flip rates at temperatures ranging from 10 to 20 °C using Equation (1). To guide the eye, dashed lines in A and C represent linear fits with zero slope and dotted lines in B are simple links of the respective data points.

Macromolecular crowders, which mimic the crowded environment in living cells, are believed to alter certain properties of proteins due to excluded volume effects and increased viscosity.^[^ ^46^ ^]^ In some cases, change of internal protein dynamics with crowding has been observed.^[^ ^47^, ^48^ ^]^ It is well conceivable that crowder molecules might have an impact on the ring‐flip dynamic in the interior of proteins, because they occupy space in the solvent that the protein needs in its expanded transition state of a ring flip.^[^ ^19^, ^20^, ^25^, ^26^, ^27^ ^]^ By this logic, an increasing crowder concentration should lower the ring‐flip rate constant. We investigated this impact with two chemically distinct small molecular crowders, polyethylene glycol (PEG)1000 and Dextran‐20, respectively. Up to a concentration of 300 g L^−1^ no change in ring‐flip rate constants and ^13^C linewidths could be observed within errors (**Figure** **4** and S3, Supporting Information). However, one observes significant performance losses, because the crowders slow down the tumbling of the protein, thereby increasing transverse relaxation (*R*_2_) and decreasing S/N. This becomes visible in the increased error bars. At 300 g L^−1^, this effect becomes so severe that relaxation dispersion experiments at 10 °C were hard to reliably quantify, if not possible. Therefore, we did not report the activation enthalpy for this condition. However, it is clear that molecular crowders do not have an impact on the ring‐flip dynamics in the interior of proteins, at least not up to concentrations which are workable for solution NMR.

### 2.4 The Internal Ring‐Flip Dynamic of a Protein is Inert to Changes in the Solvent

All the results provided earlier show that the ring‐flip dynamics in the interior of a protein—transient conformational transitions within the core of a protein visualized by aromatic ring flips—can be, and if generalized from this study is, rather inert to changes within the aqueous solvent, but depends on the makeup and the structure of a protein itself. It might be that specific interactions (e.g., binding) affect the ring‐flip dynamic, but unspecific interactions do not, or at least do not have to. In other words, in this aspect, the protein is not a slave to the solvent. These findings complement and extend observations in solids, where the crystal packing does not influence the ring‐flip dynamic.^[^ ^49^ ^]^ Furthermore, ring‐flip studies at different buffer conditions are comparable and fundamentally reported on the protein itself.

## 3 Conclusions

Here, we show that the ring‐flip dynamics of fully buried F52 of GB1 are not affected by variations of pH, salt, surface tensions, and crowding agents in the aqueous solvent. This includes not only an unchanged overall activation energy, exemplified by unaltered ring‐flip rate constants, but also unchanged activation enthalpy and entropy. The ring‐flip dynamic in the interior of GB1 therefore is an example of a fundamental dynamic process which is only affected by the surrounding protein and not by the solvent. How generally valid this finding is remains a question for future research. As a consequence, experimental ring‐flip rate constants of the same ring can be comparable among moderate deviations of buffer conditions.

## 4 Experimental Section

4.1

4.1.1

##### Protein Samples

Here, 2‐^13^C‐glucose‐labeled B1 domain of Staphylococcal protein G (GB1; UniProtKB P06654) was expressed and purified as described elsewhere,^[^ ^50^ ^]^ resulting in the site‐selective ^13^C enrichment at the ε positions of Phe and Tyr residues.^[^ ^28^, ^33^ ^]^ The sample was dissolved to a concentration of around 2 mM in 20 mM HEPES, 90% H_2_O + 10% D_2_O. PEG1000 and Dextran‐20 (average molecular mass 20,000) were purchased from Sigma‐Aldrich Chemie GmbH and from Thermo Fisher Scientific Inc., respectively. All reagents were reagent grade and used without further purification. NaI, Na_2_SO_4_, PEG1000, and Dextran‐20 solutions were prepared in 20 mM HEPES buffer. After addition of appropriate amounts to the protein solution, the pH was adjusted to 7.0 with sodium hydroxide or hydrogen chloride. In pH dependent studies, the pH was adjusted in the buffered protein solution to 6.0, 7.0, or 8.0, respectively.

##### NMR Spectroscopy

All experiments were performed on a Bruker Avance III spectrometer operating at a static magnetic field strength of 14.1 T. Aromatic L‐optimized transverse relaxation‐optimized spectroscopy (TROSY) ‐selected on‐resonance ^13^C *R*_1ρ_ relaxation dispersion experiments^[^ ^41^ ^]^ were acquired at temperatures of 10, 15, and 20 °C, if not stated otherwise.

##### Data Analysis

NMR spectra were processed with NMRPipe^[^ ^51^ ^]^ and analyzed with PINT.^[^ ^52^ ^]^ *R*_1ρ_ relaxation dispersion data were fitted to the general equation for symmetric exchange derived by Miloushev & Palmer^[^ ^53^ ^]^ using fixed populations, *p*_1_ = *p*_2_ = 0.5, and treating Δ*δ* as fixed at the value (Δ*δ*_spectra_) measured from heteronuclear single quantum coherence spectra under slow‐exchange conditions.^[^ ^19^ ^]^ Data sets acquired at a given pH, salt, or crowder concentration and different temperatures were fitted simultaneously, while imposing the restrictions: *k*_flip_(*T*_high_) > *k*_flip_(*T*_low_) and *R*_2,0_(*T*_high_) ≤ *R*_2,0_(*T*_low_).^[^ ^19^ ^]^

^13^C linewidths and errors were derived from the analysis of five spectra at each condition.

Activation parameters of the ring flips were determined by nonlinear regression of the flip rates, *k*_flip_ = *k*_ex_/2, on the temperature *T*, using the Eyring equation. The Eyring equation was parameterized as

(1) $$k_{\text{flip}}=\left(\frac{k_{B}T}{h}\right)\times \exp\left[-\left(\Delta H^{‡}-T\Delta S^{‡}\right)/RT\right]$$

where *k*_B_ and *h* are Boltzmann's and Planck's constants, respectively, and Δ*H* ^‡^ and Δ*S* ^‡^ are the activation enthalpy and activation entropy, respectively. Errors in the fitted parameters were estimated using Monte–Carlo simulations;^[^ ^54^ ^]^ the reported errors correspond to one standard deviation.

## Conflict of Interest

The authors declare no conflict of interest.

## Supporting information

Supplementary Material

## Data Availability

The data that support the findings of this study are available in the supplementary material of this article.
