# Supplementary material for: Changes in the Aqueous Solvent do not Impact the Internal Ring‐Flip Dynamic of Fully Buried F52 in Protein GB1
Source: Chembiochem. 2025 Jun 4;26(13):e202500183. doi: 10.1002/cbic.202500183 (PMC12247019; doi:10.1002/cbic.202500183)
Supplement: Supplementary file 1 — Supplementary Material [file CBIC-26-e202500183-s001.pdf]

Changes in the Aqueous Solvent do not Impact the Internal Ring Flip Dynamic of Fully Buried F52 in Protein GB1

Matthias Dreydoppel[1], Mikhail Achkinazi[1], Charlotte Krünholz[1], Paula L. Jordan[1,2] and Ulrich Weininger[1,*]

[1] Institute of Physics, Biophysics, Martin-Luther-University Halle-Wittenberg, D-06120 Halle (Saale), Germany

[2] Department of Radiology, Medical Physics, University Medical Center Freiburg, Faculty of Medicine, University of Freiburg, D-79106 Freiburg, Germany

* Correspondence:

email: ulrich.weininger@physik.uni-halle.de

phone: +49 345 55 28555

fax: +49 345 55 27161

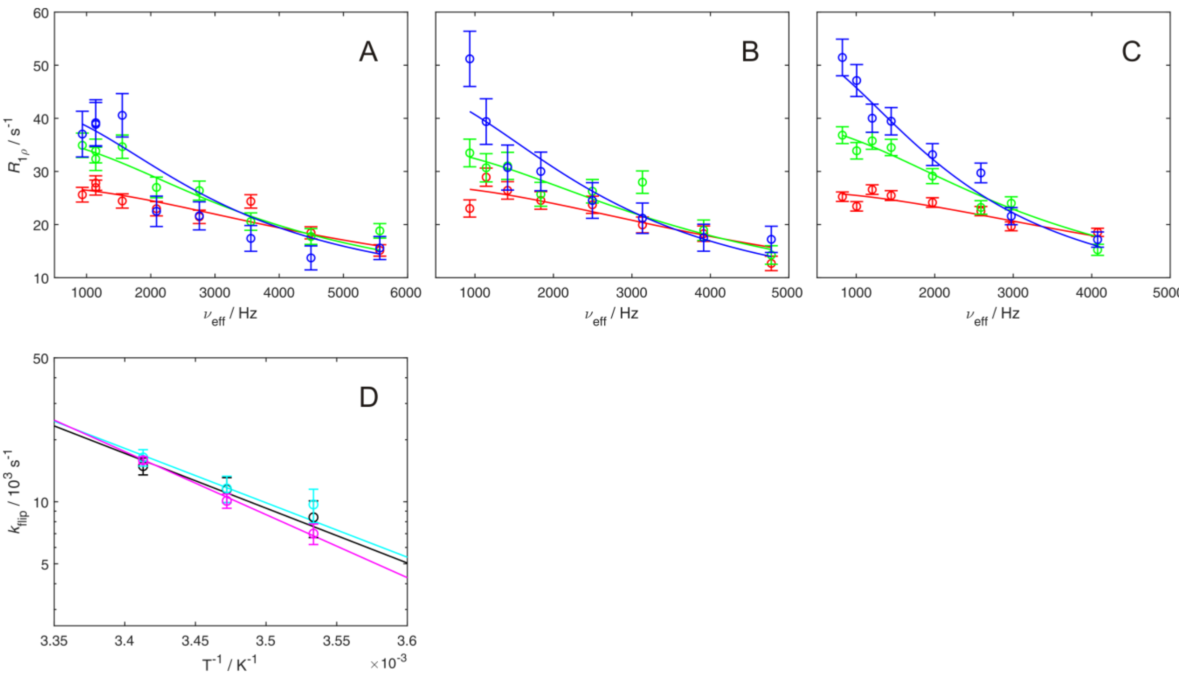

Figure S1: (A-C) ^{13}C aromatic $R_{1\rho}$ relaxation dispersion profiles for F52ε recorded on-resonant ($\theta > 85°$) on a 2 mM sample of GB1 at a static magnetic field strength of 14.1 T and pH values of 6.0 (A), 7.0 (B), and 8.0 (C). The temperatures at each pH value were set to 10 °C (blue), 15 °C (green) and 20 °C (red). The relaxation dispersions were fitted using a fixed population $p_1 = p_2 = 0.5$ and $\Delta\delta$ fixed at the value measured from HSQC spectra under slow-exchange conditions, with the restrictions: k_{flip} (T_{high}) > k_{flip} (T_{low}), $R_{2,0}$ (T_{high}) ≤ $R_{2,0}$ (T_{low}). (D) Temperature dependence of flip rates. k_{flip} is plotted as a function of 1/T at pH values of 6.0 (cyan), 7.0 (black), and 8.0 (magenta). The fits of eq. 1 to the data are displayed as lines. The data are represented using a logarithmic y-axis to show the expected linearity, but the fit was performed using non-linear regression of k_{flip} on T.

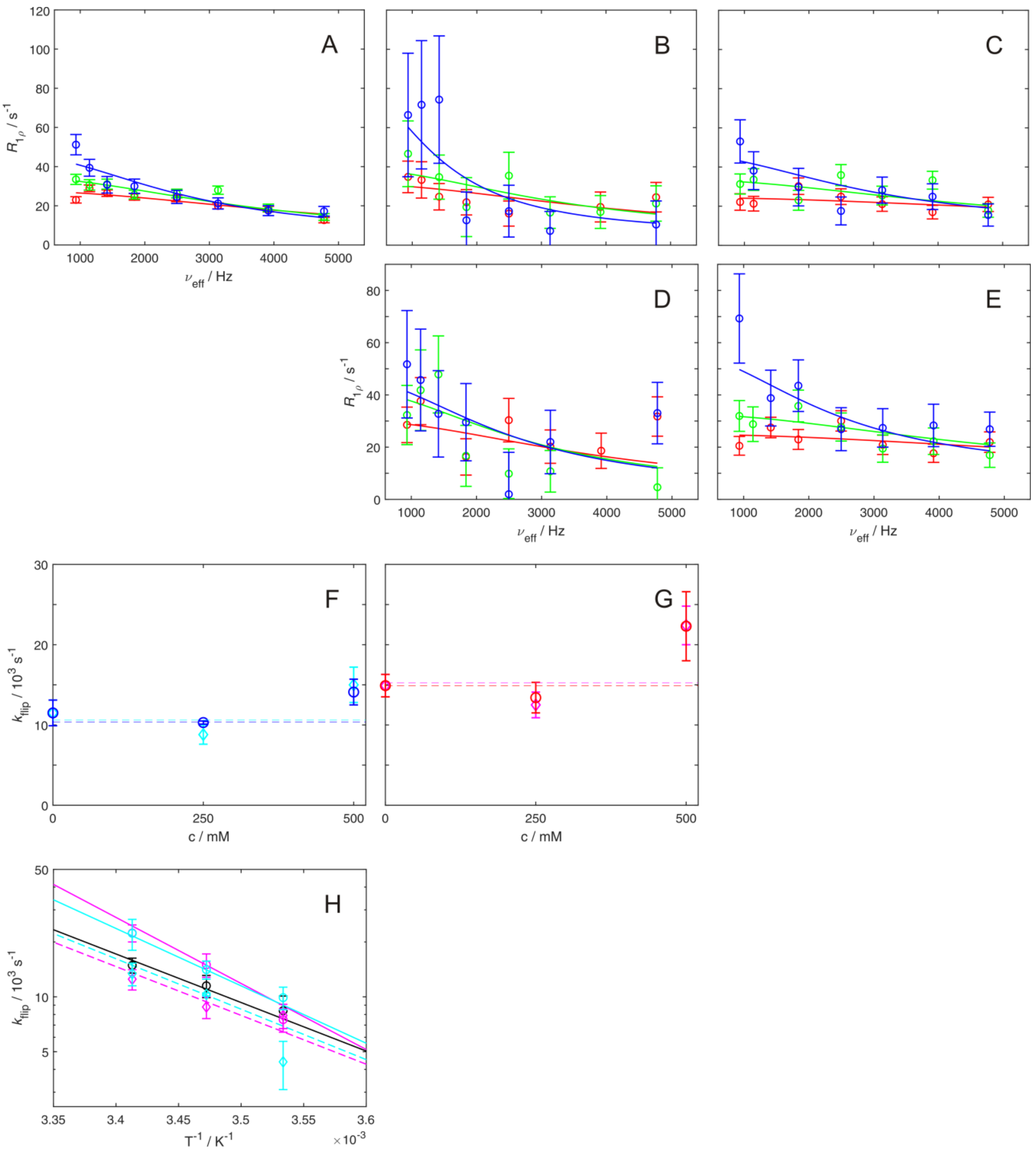

Figure S2: (A-E) ^{13}C aromatic $R_{1\rho}$ relaxation dispersion profiles for F52ε recorded on-resonant ($\theta > 85°$) on a 2 mM sample of GB1 at a static magnetic field strength of 14.1 T, pH 7.0 and different salt concentrations. (A) Without salt, (B) 250 mM NaI, (C) 500 mM NaI, (D) 250 mM Na_2SO_4, and (E) 500 mM Na_2SO_4. The temperatures were set to 10 °C (blue), 15 °C (green) and 20 °C (red). The relaxation dispersions were fitted using a fixed population $p_1 = p_2 = 0.5$ and $\Delta\delta$ fixed at the value measured from HSQC spectra under slow-exchange conditions, with the restrictions: k_{flip} (T_{high}) > k_{flip} (T_{low}), $R_{2,0}$ (T_{high}) ≤ $R_{2,0}$ (T_{low}). (F-G) Salt concentration dependence of ring flip rates. k_{flip} is plotted as a function of the concentration of NaI and of Na_2SO_4, at temperatures of 15 °C (F, blue and cyan, respectively) and 20 °C (G, red and magenta, respectively). (H) Temperature dependence of flip rates. k_{flip} is plotted as a function of $1/T$ without salt (black), with 250 mM and 500 mM NaI (cyan, with dotted and solid lines, respectively), with 250 mM and 500 mM Na_2SO_4 (magenta, with dotted and solid lines, respectively). The fits of eq. 1 to the data are displayed as lines. The data are represented using a logarithmic y-axis to show the expected linearity, but the fit was performed using non-linear regression of k_{flip} on T.

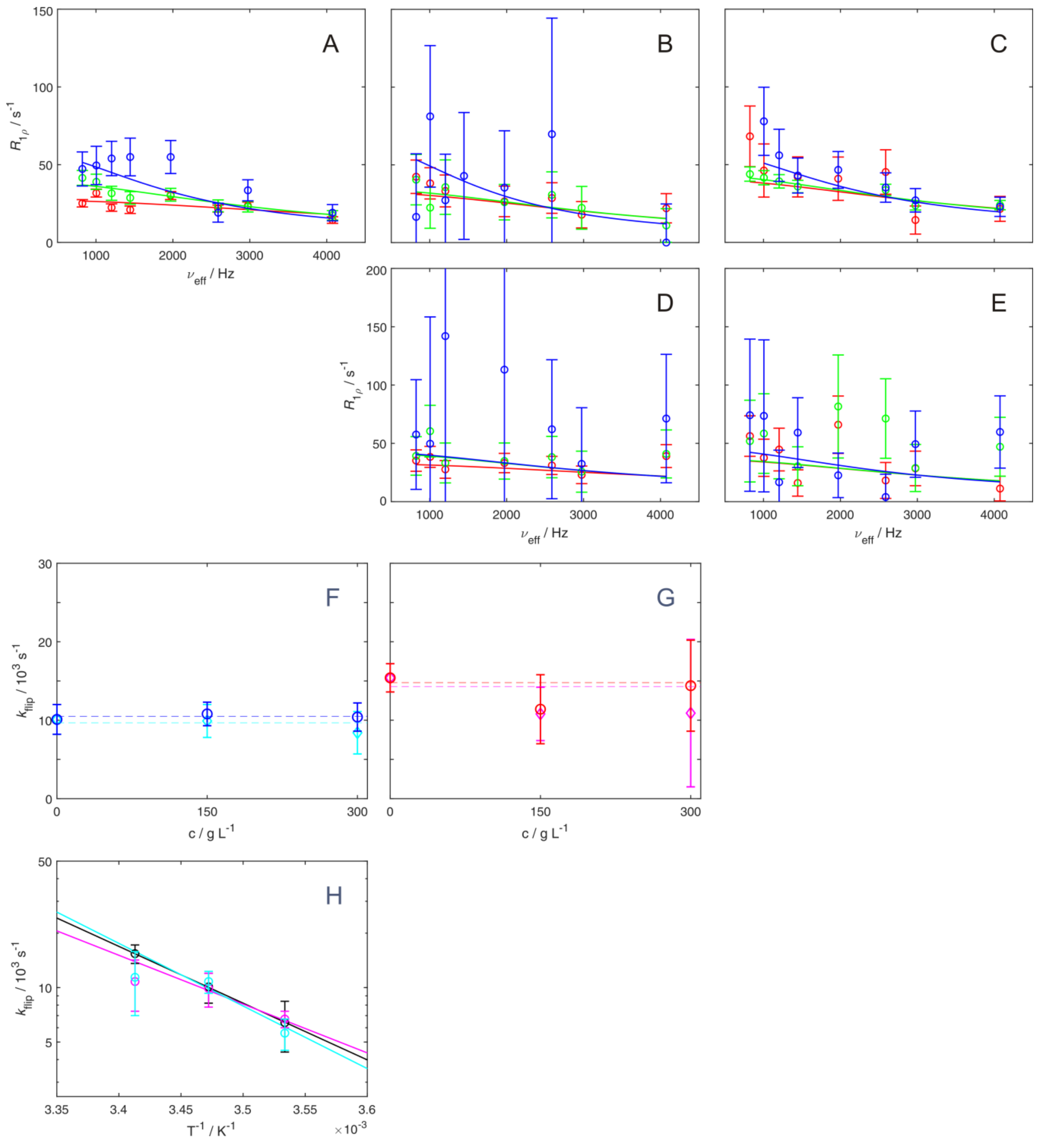

Figure S3: (A-E) ^{13}C aromatic $R_{1\rho}$ relaxation dispersion profiles for F52ε recorded on-resonant ($\theta > 85°$) on a 2 mM sample of GB1 at a static magnetic field strength of 14.1 T, pH 7.0 and different crowder concentrations. (A) Without crowders, (B) 150 g/L PEG, (C) 150 g/L dextran, (D) 300 g/L PEG, and (E) 300 g/L dextran. The temperatures are: (A-C) 10, 15 and 20 °C, (D) 13, 15 and 20 °C, and (E) 15, 17 and 20 °C, in blue, green and red, respectively. The relaxation dispersions were fitted using a fixed population $p_1 = p_2 = 0.5$ and $\Delta\delta$ fixed at the value measured from HSQC spectra under slow-exchange conditions, with the restrictions: k_{flip} (T_{high}) > k_{flip} (T_{low}), $R_{2,0}$ (T_{high}) ≤ $R_{2,0}$ (T_{low}). (F-G) Crowder concentration dependence of ring flip rates. k_{flip} is plotted as a function of the concentration of PEG and of dextran at temperatures of 15 °C (F, blue and cyan, respectively) and 20 °C (G, red and magenta, respectively). (H) Temperature dependence of flip rates. k_{flip} is plotted as a function of $1/T$ without crowders (black), with 150 g/L PEG (cyan), with 150 g/L dextran (magenta). The fits of eq. 1 to the data are displayed as lines. The data are represented using a logarithmic y-axis to show the expected linearity, but the fit was performed using non-linear regression of k_{flip} on T.
